# Association of oral anticoagulants with risk of brain haemorrhage expansion compared to no-anticoagulation

**DOI:** 10.1186/s42466-024-00358-9

**Published:** 2025-02-12

**Authors:** Roland Veltkamp, Kirsten Haas, Viktoria Rücker, Uwe Malzahn, Adrian Heeger, David Kinzler, Patrick Müller, Pascal Rappard, Timolaos Rizos, Johannes Schiefer, Christian Opherk, Waltraud Pfeilschifter, Katharina Althaus, Peter Schellinger, Bernadette Gaida, Maria Magdalena Gabriel, Georg Royl, Darius G. Nabavi, Karl Georg Haeusler, Christian H. Nolte, Marc E. Wolf, Sven Poli, Marilen Sieber, Pascal Mosimann, Peter U. Heuschmann, Jan C. Purrucker, on behalf of the RASUNOA-prime investigators, Solveig Horstmann, Alexandra Krauß, Caroline Renninger, Peter Ringleb, Arno Reich, Eve Kohler, Erendira Boss, Jan Hendrik Schaefer, Marc Pflug, Bettina von Sarnowski, Gerrit Maximilian Große, Johanna Ernst, Karin Weißenborn, Ramona Schupper, Hans Worthmann, Susanne Riebau, Olaf Crome, Boris Dimitrijeski, Jens Offermann, Ida Rangus, Elisabeth Schmid, Khouloud Poli, Johannes Tünnerhoff, Dominik Michalski, Johann O. Pelz, Martin Juenemann, Tobias BraunTobias J. Müller, Katja Wartenberg, Andreas Binder, Johannes Meyne, Benno Ikenberg, Johanna Härtl, Michael Ohms, Sebasitan Edelbusch, Marc Fatar, Angelika Alonso, Martin Nückel, Frank Erbguth, Alexandra Grau, Udo Selig, Rainer Dziewas, Jens Minnerup, Kristian Barlinn, Kathrin Haase, Götz Thomalla, Milani Deb-Chatterji, Mathias Mäurer, Mathias Pfau, Peter Michels, Zoran Vukovic, Timo Uphaus, Klaus Gröschel, Sonja Gröschel, Marianne Hahn, Joannes Mühler, Klaus Dötter, Michaela Wagner-Heck, Frank Arne Wollenweber, Sebastian Jander, John-Ih Lee, Wolf-Rüdiger Schäbitz, Inken Piehl, Michael Frattner, Dimitre Staykov, Christian Urbanek, Sabine Schröder, Sylke Düllberg-Boden, Pawel Kermer, Matthias Kaste, Lars Marquardt, Haiko Kazarians, Christoph Kleinschnitz, Peter Kraft, Frank Hoffmann, Andrea Kraft, Jürgen Hartmut Faiss, Gernot Reimann, Michael Schwarz, Thorsten Steiner, Albrecht Günther, Uta Meyding Lamadé, Matthias W. Lorenz

**Affiliations:** 1 Department of Brain Sciences, Imperial College London, London, UK; 2 Department of Neurology, Alfried-Krupp Hospital, Essen, Germany; 3 Department of Neurology, Heidelberg University Hospital, Heidelberg, Germany; 4 Institute of Clinical Epidemiology and Biometry, Julius-Maximilians-Universität Würzburg (JMU), Würzburg, Germany; 5 Clinical Trial Center, University Hospital Würzburg, Würzburg, Germany; 6 Department of Neurology, University Hospital RWTH Aachen, Aachen, Germany; 7 Department of Neurology, SLK-Kliniken Heilbronn, Heilbronn, Germany; 8 Department of Neurology, University Hospital Frankfurt, Goethe-University, Frankfurt Am Main, Germany; 9 Department of Neurology, Klinikum Lüneburg, Lüneburg, Germany; 10 Department of Neurology, University Hospital Ulm, Ulm, Germany; 11 Department of Neurology and Neurogeriatrics, Johannes Wesling University Hospital Minden, Minden, Germany; 12 Universitätsklinikum Ruhr-Universität Bochum, Bochum, Germany; 13 Department of Neurology, University Medicine Greifswald, Greifswald, Germany; 14 Department of Neurology, Hannover Medical School, Hannover, Germany; 15 Neurovascular Center, University Hospital Schleswig-Holstein, Campus Lübeck, Lübeck, Germany; 16 Vivantes Klinikum Neukölln, Berlin, Germany; 17 Department of Neurology, Universitätsklinikum Würzburg (UKW), Würzburg, Germany; 18 Center for Stroke Research Berlin (CSB), Berlin, Germany; 19 Department of Neurology With Experimental Neurology, Berlin Institute of Health (BIH), Charité Universitätsmedizin Berlin, Berlin, Germany; 20 Department of Neurology, Klinikum Stuttgart, Stuttgart, Germany; 21 Department of Neurology & Stroke, Tübingen University Hospital, Tübingen, Germany; 22 Hertie Institute for Clinical Brain Research, Tübingen University Hospital, Tübingen, Germany; 23 Department of Neuroradiology, Toronto Western Hospital Division of Neuroradiology, Toronto, Canada; 24 Clinical Trial Center Würzburg, University Hospital Würzburg, Würzburg, Germany; 25 Institute for Medical Data Science, University Hospital Würzburg, Würzburg, Germany

## Abstract

**Background:**

The impact of direct oral anticoagulants (DOAC) on haematoma size after intracerebral haemorrhage (ICH) compared to no-anticoagulation is controversial and prospective data are lacking.

**Methods:**

The investigator-initiated, multicentre, prospective RASUNOA-prime study enrolled patients with non-traumatic ICH and atrial fibrillation while on a DOAC, vitamin K antagonist (VKA) or no anticoagulation (non-OAC). Neuroimaging was reviewed centrally blinded to group allocation. Primary endpoint was haematoma expansion (≥ 6.5 ml or ≥ 33%, any new intraventricular blood or an increase in modified Graeb score by ≥ 2 points) between baseline and follow-up scan within 72 h after symptom onset.

**Results:**

Of 1,440 patients screened, 951 patients with ICH symptom onset less than 24 h before admission were enrolled. Baseline scans were performed at a median of 2 h (IQR 1–6) after symptom onset. Neurological deficit and median baseline haematoma volumes (11 ml; IQR 4–39) did not differ among 577 DOAC, 251 VKA and 123 non-OAC patients. Haematoma expansion was observed in DOAC patients in 142/356 (39.9, 95%-CI 34.8–45.0%), VKA in 47/155 (30.3, 95-CI 23.1%–37.6%), versus non-OAC in 22/74 (29.7, 19.3–40.1%). Unspecific reversal agents in DOAC-ICH (212/356, 59.6%) did not affect the haematoma expansion rate compared to no-antagonization.

**Conclusion:**

Baseline haematoma volume and risk of haematoma expansion did not differ statistically significantly in patients with and without DOAC.

**Supplementary Information:**

The online version contains supplementary material available at 10.1186/s42466-024-00358-9.

Members listed in supplement

## Introduction

Intracranial haemorrhage develops in 0.07 to 0.5% of patients anticoagulated with a direct oral anticoagulant (DOAC) annually [1], which results in death in 24 to 67% of patients and significant disability in intracerebral haemorrhage (ICH) survivors [2–6]. Haematoma size is a key prognostic factor in ICH [7]. A therapeutic target after ICH in general and in OAC-related ICH in particular is to prevent early haematoma expansion [8, 9]. Although solid evidence from prospective studies regarding the putative excess risk of haematoma expansion in DOAC-ICH compared to no anticoagulation is missing, current guidelines—in analogy to vitamin K antagonists (VKA)-ICH—recommend that management of DOAC-ICH should comprise anticoagulant reversal [10, 11]. The specific reversal agents idarucizumab and andexanet alfa have been licensed for reversing the anticoagulant effect of dabigatran and two factor Xa inhibitors, respectively. Recently, for andexanet alfa a reduction of haematoma expansion rates compared to a group receiving mostly prothrombin complex concentrate (PCC) was shown in a randomized trial [12]. However, detailed evidence regarding the characteristics of DOAC-ICH in comparison to non-anticoagulated atrial fibrillation (AF) patients with ICH from prospective studies in clinical routine is needed to further understand the characteristics of haematoma expansion and the potential usefulness of haemostatic measures in acute DOAC-ICH.

The aim of our prospective multicentre study was to describe the characteristics of ICH in AF patients receiving one out of three different types of treatment at the time of the ICH: (1) DOAC, (2) VKA or (3) no anticoagulation. We hypothesized that, in terms of substantial haematoma expansion rates, DOAC treatment before ICH is not inferior to no anticoagulation in AF patients experiencing ICH.

## Methods

### Study design and selection criteria

The Registry of Acute Stroke under Novel Oral Anticoagulants-Prime (RASUNOA-prime) was a prospective, multicentre, observational registry study of patients with AF and acute ischemic stroke or ICH (clinicaltrials.gov, NCT02533960). The ethics committee of the Medical Faculty of the University Heidelberg as well as all local ethics committees approved the study protocol (S-088/2015). Details of the study design have been published [13]. Herein, we report on the pre-specified study of patients with ICH. The study was conducted between 2015 and 2021 in 46 certified German and Austrian Stroke Units where consecutive patients with spontaneous non-traumatic ICH and AF were enrolled. Patients were eligible if they were 18 years or older and either the patient or a legal representative provided consent. Informed consent could be waived if patients were not able to consent and died before a legal representative could be installed. Exclusion criteria encompassed symptom onset of more than 24 h before admission, traumatic ICH and treatment with a parenteral anticoagulant.

Three groups were studied: (1) patients with evidence of intake of a DOAC (apixaban, dabigatran, edoxaban or rivaroxaban), (2) patients taking a VKA, and (3) patients not taking an anticoagulant (non-OAC) at the time of the ICH. Non-OAC included patients in whom prescription of a DOAC or VKA had been discontinued and in whom the last dose of a DOAC was given ≥ 72 h or a VKA ≥ 7 days before admission, respectively.

To ensure that all three treatment arms are equally distributed at any time of recruitment, reflecting potentially changing clinical practice and recommendations, enrolment followed a standardised algorithm in which each site had to recruit an ICH patient on a DOAC first and only subsequently was allowed to enrol patients into the two other study arms. Hereby, potential changes in the treatment of ICH patients over time is taken into account by obtaining controls (VKA or no-anticoagulation) each time a case (DOAC) is enrolled [13]. Three-month survival was ascertained either by a questionnaire, a phone call to patients or their designated representatives or by contacting the registration office.

### Neuroimaging analysis

Each brain scan (computed tomography [CT] or magnetic resonance imaging [MRI]) was assessed using Osirix Pro software (Version 12 or newer) at the central imaging core laboratory by two trained, independent reviewers, and in case of disagreement, by a third reviewer, all of whom were blinded to group allocation. The analysis followed a pre-specified imaging analysis manual. Haematoma volume was measured by planimetry after reviewers had encircled intracerebral haematomas, excluding intraventricular blood (IVH) (as done previously [3]). To capture the entire spectrum of ICH encountered in clinical routine, follow-up brain imaging was not a mandatory study procedure because additional radiation exposure would have required prior consent. The development of haematoma volume was assessed on all available follow-up CTs or MRIs that had been performed within 72 h of symptom onset. The upper time limit was chosen as relevant residual DOAC activity is considered unlikely even in patients with renal failure 72 h after last intake. The modified Graeb score was calculated [14]. Substantial haematoma expansion was defined if haematoma volume on follow-up scans exceeded the baseline haematoma volume by ≥ 6.5 ml or ≥ 33%, or new intraventricular haemorrhage was found or intraventricular haematoma increased by ≥ 2 points on the mGraeb scale. Images were evaluated for haematoma expansion if no surgical haematoma evacuation or external ventricular drainage was evident before the respective follow-up scan.

### Statistical analysis

The statistical analysis followed a pre-specified statistical analysis plan and was performed according to the Strengthening of the Reporting of Observational Studies in Epidemiology (STROBE) guidelines for observational studies. Although the study was observational, a primary hypothesis was made that the proportion of haematoma expansion in DOAC-ICH is not inferior to ICH without prior OAC. Sample size estimates were made based on the assumption that in non-OAC ICH patients, the rate of relevant haematoma expansion is approximately 28% [13]. We considered a clinically relevant increased risk of haematoma expansion if the rate in DOAC-ICH was more than 10% above this rate. To detect non-inferiority by a one-sided unpooled two-sample z-test with an alpha of 2.5%, a power of 80% and assuming a drop-out rate of 5%, 333 patients were needed per group. The recruitment strategy was modified in October 2019 after N = 651 patients had been enrolled and it had become clear that the originally planned sample size distribution per group could not be reached because ICH in patients with AF occurred much more frequently while receiving a DOAC or a VKA than in patients not receiving anticoagulants. Thereafter, recruitment was continued until nearly 1000 ICH patients were enrolled, irrespective of group allocation.

As no comparison of VKA and DOCA was intended, DOAC and VKA were compared against non-OAC separately. Comparison of two groups (DOAC vs. non-OAC or VKA vs. non-OAC) and exploratory analysis of subgroups (with/without radiological follow-up; anticoagulation reversal within DOAC; within VKA-ICH) was conducted by applying the Chi^2^-test or t-Test or Mann–Whitney U Test, according to the corresponding data measurement scale and properties of the observed distributions. The analysis of the primary outcome was a test of non-inferiority (Farrington-Manning score test) at significance level 2.5% using the pre-defined non-inferiority margin of 10%. After that a further non-inferiority test comparing VKA vs. non-OAC was conducted, using the same margin (secondary analysis). For all further analyses, the significance level was set to 5%. To include factors potentially affecting haematoma expansion rates the comparison was adjusted using stepwise modelling. In a first step, univariable and multivariable logistic regression (model 1) for haematoma expansion adjusting for pre-defined confounders (age, onset to brain imaging in hours, baseline haematoma volume, systolic blood pressure, concomitant antiplatelet therapy, ICH localization and pre-stroke mRS score (0–3 vs. 4–5, not documented) without the factor anticoagulation scheme at index event was conducted. The model-predicted logit (multivariable) of the event was used as a risk score in the subsequent analysis. In a second step, to compare DOAC vs. non-OAC and VKA vs. non-OAC separately, two logistic regression analyses were performed (model 2), each adjusted for the risk score out of the resulting model in step 1. A sensitivity analysis for the primary endpoint counting patients with early death (within the first day) as having the primary endpoint was also analysed. A second sensitivity analysis for the primary endpoint regarding effects of sites using generalized linear mixed models adjusted for random effects of centres was conducted.

## Results

Out of 1,440 screened patients, 951 met the eligibility criteria and formed the “baseline cohort”.

Main characteristics of the baseline cohort are shown in Table 1 and additional characteristics in the online supplementary table S1. Overall, mean age was 79.1 (± 8.1) years and 45.1% were female. No differences were present among groups except for type of AF, a less frequent history of previous ICH in DOAC (3.5%) and VKA (2.0%) vs. non-OAC patients (10.5%), and a higher rate of antiplatelet therapy in non-OAC patients. Furthermore, pre-stroke mRS was lower in the VKA group compared to non-OAC. At hospital admission, neurological deficit and disability scores did not differ between anticoagulated and non-anticoagulated patients.

**Table 1:** Main characteristics of the ‘baseline cohort’ by anticoagulation scheme

	DOAC (N = 557)		VKA (N = 251)		Non-OAC (N = 123)		*p* values	
Variable	N	Value	N	Value	N	Value	DOAC vs. non-OAC	VKA vs. non-OAC
Age in years, mean (SD)	577	79.2 (8)	251	79.3 (7.5)	123	78.0 (9.5)	0.216	0.184
Female sex, n (%)	577	277 (48)	251	95 (37.8)	123	57 (46.3)	0.737	0.116
CHA2DS2VASc, median (IQR)	573	4 (3–6)	250	4 (3–5)	121	4 (3–5)	0.013	0.339
HAS-BLED, median (IQR)	572	2 (2–3)	246	2 (2–3)	121	2 (2–3)	0.138	0.716
Antiplatelet therapy, n (%)	567	64 (11.3)	247	21 (8.5)	118	53 (44.9)	< 0.0001	< 0.0001
SBP at admission, mmHg, median (IQR)	537	170 (150–190)	235	167 (149–190)	116	163 (149–200)	0.748	0.830
SBP at 24 h, mmHg, median (IQR)	423	138 (120–150)	187	130 (120–148)	105	135 (120–150)	0.705	0.401
Reversal treatment, n (%)								
None	577	239 (41.4)	251	58 (23.1)				
PCC	577	288 (49.9)	251	193 (76.9)				
Specific†	577	50 (8.7)						
Surgical treatment, n (%)								
Haematoma evacuation	577	26 (4.5)	251	16 (6.4)	123	4 (3.3)	0.533	0.207
Ventricular drainage	577	51 (8.8)	251	29 (11.6)	123	9 (7.3)	0.584	0.203
NIHSS at admission, median (IQR)	545	10 (4–20)	238	10 (4–20)	122	9 (4–18)	0.680	0.497
modified Rankin scale score, median (IQR)								
Pre-stroke	506	1 (0–3)	217	1 (0–2)	104	1 (0–3)	0.192	0.019
At admission	562	4 (3–5)	249	4 (3–5)	121	4 (3–5)	0.486	0.882
Discharge	561	5 (3–6)	245	4 (3–6)	120	4 (3–5)	0.171	0.648
Death during acute stay, n (%)	577	174 (30.2)	251	74 (29.5)	123	28 (22.8)	0.101	0.171
Mortality at 3 months, n (%)	492	249 (50.6)	218	101 (46.3)	101	44 (43.6)	0.197	0.644

CHA2DS2VASc, Cardiac Failure or Dysfunction; Hypertension, Age ≥ 75 years (Doubled), Diabetes, Stroke (Doubled)–Vascular Disease, Age 65–74 Years, and Sex Category (Female); DOAC, direct oral anticoagulant; HAS-BLED, Hypertension; Abnormal Renal/Liver Function, Stroke, Bleeding History or Predisposition, Labile INR [international normalized ratio], Elderly, Drugs/Alcohol Concomitantly; NIHSS, National Institutes of Health Stroke Scale; Non-OAC, no oral anticoagulation; PCC, prothrombincomplex concentrate; SBP, systolic blood pressure; VKA, vitamin K antagonists

^†^ Dabigatran: idarucizumab, Factor Xa-inhibitors: andexanet alfa

The first brain scan was performed at a median of 2 h (IQR 1–6) after symptom onset. Median baseline haematoma volume did not differ significantly among groups at this early time point (Table 2). Overall, any ventricular haemorrhage had developed in 43.3% of patients. No significant differences among groups were noted regarding median length of hospital stay, in-hospital mortality, and mortality at 3 months (Tables 1 and S1).

**Table 2:** Radiological characteristics of the ‘baseline cohort’ by anticoagulation scheme

	DOAC (N = 577)		VKA (N = 251)		Non-OAC (N = 123)		*p* values	
Variable	N	Value	N	Value	N	Value	DOAC vs. non-OAC	VKA vs. non-OAC
Onset to first brain imaging‡, hours, median (IQR)	532	2 (1–6)	237	2 (1–6)	109	3 (1–7)	0.087	0.444
Baseline haematoma volume, mL, median (IQR)	556	13 (4–38)	247	11 (5–43)	120	10 (3–30)	0.344	0.220
Ventricular extension, n (%)	577	252 (43.7)	251	115 (45.8)	123	45 (36.6)	0.149	0.090
Surgical treatment, n (%)								
Haematoma evacuation	577	26 (4.5)	251	16 (6.4)	123	4 (3.3)	0.533	0.207
Ventricular drainage	577	51 (8.8)	251	29 (11.6)	123	9 (7.3)	0.584	0.203

DOAC, direct oral anticoagulant; No-OAC, no oral anticoagulation; VKA, vitamin K antagonists

^‡^ Onset: symptom onset, in case of unknown onset, last-seen-well

### Haematoma expansion

**Fig. 1:**
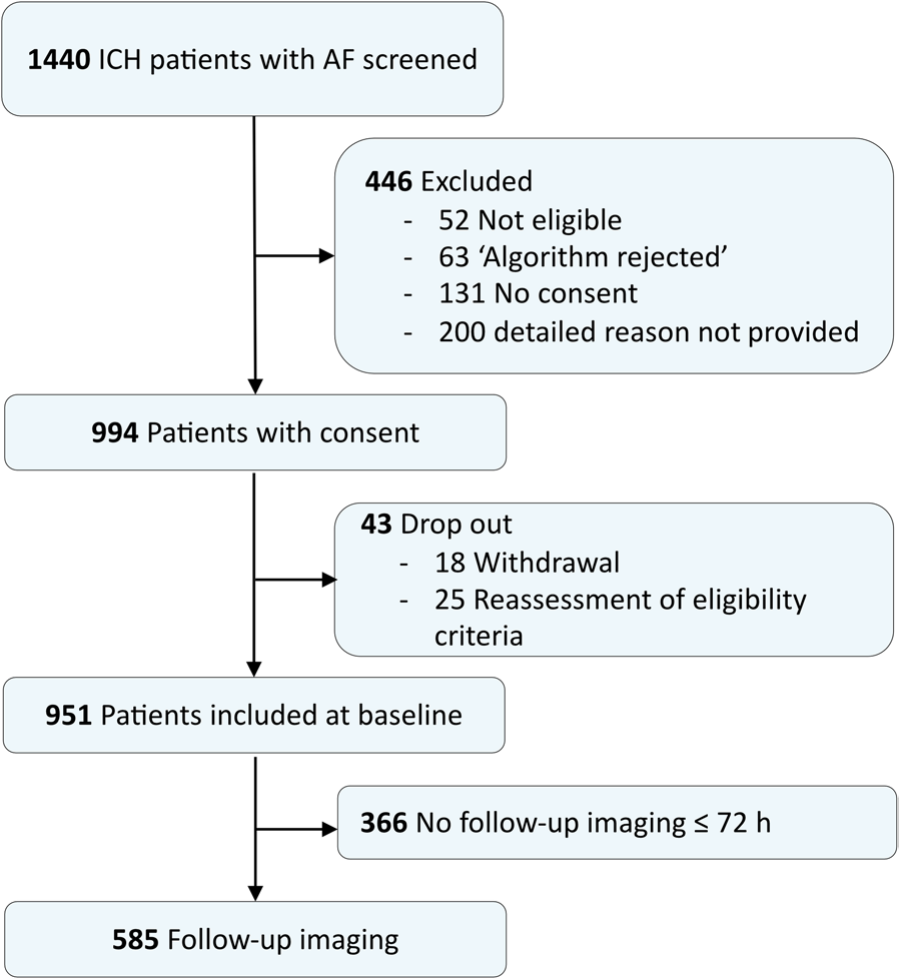
Flow chart of patient enrolment

The “haematoma expansion analysis cohort (HE-cohort)” comprised 585 patients with available follow-up scans within 72 h after symptom-onset and no early intracranial surgical treatment. Decisions not to perform follow-up imaging was made by local physicians including decisions for early palliative care (49%; Table S2). Figure 1 illustrates the patient flow.

Compared to patients without radiological follow-up patients in the ‘HE-cohort’ were younger, had a lower NIHSS score at admission and the baseline haematoma volume was smaller (suppl. Table S2). No differences were present among groups except that the non-anticoagulated group included more patients with previous ICH (suppl. Table S3).

Substantial haematoma expansion within 72 h after symptom onset developed in 142 (39.9, 95%-CI 34.8–45.0%) of DOAC patients, in 47 (30.3%, 95%-CI 23.1%–37.6%) of VKA patients and in 22 (29.7, 19.3–40.1%) of patients without anticoagulation (Table 3). Non-inferiority of the proportion of patients with haematoma expansion between DOAC and non-anticoagulated patients, as well as between VKA and non-anticoagulated patients was not met (DOAC vs. non-OAC, risk difference 10.2%, 95%-CI -1.4%–21.8%, p = 0.51; VKA vs. non-OAC, risk difference 0.6%, 95%-CI -11.6%–12.8%, *p* = 0.066, resp.).

**Table 3:** Radiological characteristics of the ‘haematoma expansion analysis cohort’

	DOAC (N = 356)		VKA (N = 155)		Non-OAC (N = 74)		*p* values	
Variable	N	Value	N	Value	N	Value	DOAC vs. non-OAC	VKA vs. non-OAC
Baseline haematoma volume, mL, median (IQR)	343	9 (3–25)	153	9 (4–23)	72	9 (3–28)	0.954	0.694
Ventricular extension, n (%)	356	140 (39.3)	155	67 (43.2)	74	24 (32.4)	0.267	0.119
Substantial haematoma expansion, n (%)	356	142 (39.9)	155	47 (30.3)	74	22 (29.7)	#	#
Haematoma expansion, absolute (mL), median (IQR)	356	0.6 (0.15–3.5)	155	0.5 (-0.5–2.6)	74	0.6 (-0.3–3.1)	0.583	0.812
De novo ventricular extension, n (%)	216	43 (19.9)	88	9 (10.2)	50	7 (14.0)	0.335	0.506

^#^ no *p* value given as non-inferiority of DOAC intake vs no anticoagulant treatment was tested, VKA intake vs no anticoagulant treatment respectively

### Factors associated with haematoma expansion

Factors associated with haematoma expansion were analysed in exploratory univariable and multivariable logistic regression models (suppl. Table S4). Longer onset to first brain imaging led to a lower risk of haematoma expansion, while larger baseline haematoma was associated with a higher risk of haematoma expansion. Higher baseline glucose levels increased the risk of haematoma expansion. No such association was found for age, pre-stroke disability, high systolic blood pressure (> 160 mmHg) at admission, concomitant antiplatelet therapy or deep versus lobar location of the ICH (model 1). In model 2 after adjusting for confounders (logits of model 1), no statistically significant association of DOAC vs. non-OAC (adj. OR 1.58, 95% CI 0.13–3.08, *p* = 0.180) or VKA vs. non-OAC (adj. OR 1.02, 95% CI 0.48–2.21, *p* = 0.952; two separate models) was found regarding the risk of haematoma expansion.

To estimate the effect of early death a sensitivity for early death was conducted, where all early deaths were counted as substantial haematoma expansion, if no follow-up imaging was available before death. This sensitivity analysis revealed higher risk differences of substantial haematoma expansion between DOAC vs. non-OAC of 13.4% (DOAC rate: 48.4%, non-OAC rate: 35.0%) and the risk difference between VKA vs. non-OAC increases to 6.6% (VKA: 41.6%). Non-inferiority was still not significant for both comparisons. However the adjusted odds ratios for DOAC vs. no-OAC (1.71 (95%-CI: 0.9–3.25) and VKA vs. no-OAC (1.21 (95%-CI: 0.60–2.47)) were similar to those from the original analysis.

Furthermore, a sensitivity analysis on the influence of centre differences revealed similar adjusted odds ratios and, therefore, no influence of the enrolling sites on the primary endpoint was evident.

### Effect of anticoagulation reversal in DOAC-ICH

We performed an exploratory analysis of the effect of different methods of anticoagulation reversal in patients treated with DOACs (suppl. Table S5). Of the 356 DOAC-ICH patients, 107 (30.1%) received no antagonization, 212 (59.6%) received 4-factor PCC, and 37 (10.4%) received a specific reversal agent (idarucizumab or andexanet alfa). At baseline, median haematoma volumes were 6.1 mL (IQR 2.1–23.5) in the group without antagonization, 9.7 mL (IQR 4.5–27.5) in the group receiving PCC and 11.3 mL in patients receiving a specific reversal treatment (IQR 2.6–18.0) (*p* = 0.08). Haematoma expansion developed in 41 patients (38.3% (95%-CI: 29.1–47.5) without antagonization, in 89 (42.0% (95%-CI: 35.4–48.6) receiving PCC and in 12 (32.4% (95%-CI: 17.4–47.5)) receiving a specific reversal agent (*p* = 0.51).

### Effect of anticoagulation reversal in VKA-ICH

Among VKA patients in the ‘HE-cohort’, ICH occurred in 72 (48.3%) within the therapeutic INR range. N = 133 (89%) received PCC in addition to standard treatment with vitamin K. Haematoma expansion occurred in 43 (31.2% (95%-CI: 23.4–38.9)) PCC-patients vs. in 4 (23.5% (95%-CI: 3.4–43.7)) patients with no PCC (*p* = 0.518). Despite a higher percentage of patients with mRS > 2 at admission in the PCC group, mortality at hospital discharge was similar between groups (suppl. Table S6).

## Discussion

The main findings are that: (1) the neurological deficit at hospital admission and the baseline haematoma volume did not differ among non-anticoagulated and anticoagulated ICH patients with AF; (2) the risk of haematoma expansion within 72 h was 10.2% higher in patients taking a DOAC at the time of ICH than in patients not taking an anticoagulant, albeit with overlapping confidence intervals; (3) in a cohort of VKA-ICH with a high proportion of anticoagulation reversal with PCC, the rate of haematoma expansion was similar to that in patients not receiving anticoagulation; (4) No trend towards a beneficial effect of PCC on haematoma expansion was observed in DOAC-ICH; and (5) in hospital and 3-month mortality rates did not differ significantly between patients receiving or not receiving an oral anticoagulant.

Previous studies [15–18] and systematic reviews with meta-analyses [2, 19] reported controversial findings regarding baseline haematoma size and clinical presentation in OAC-related ICH. In one meta-analysis including 19 studies, baseline haematoma volume was significantly larger in VKA-ICH than in non-anticoagulated patients but VKA patients were significantly older [19]. Data for comparison of DOAC with no anticoagulation were insufficient [19]. A recent analysis of two European national stroke registries reported similar adjusted lower odds of a favourable outcome for VKA and DOAC-associated ICH compared to no oral anticoagulation [20]. Both anticoagulation schemes were also associated with a higher odds of mortality. Mortality at 3-months were similar to our findings for orally anticoagulated patients, but mortality in non-anticoagulated patients differed (30% versus 44% in our population). Reasons could be the lower case-severity at admission (median NIHSS 7 versus 9), as well as the inclusion of early deceased patients and patients not capable to give consent on their own in our study. Table S7 in the supplement summarizes previous studies reporting baseline haematoma volumes or haematoma expansion rates in patients with or without oral anticoagulation. Remarkably, most of the studies that reported imaging data were retrospective or did not include a central imaging analysis. Moreover, the focus of most studies was to compare ICH patients taking a DOAC vs. a VKA rather than comparing them to patients with no anticoagulation although this comparison is best suited to characterize a putative worsening effect of DOACs on haematoma size.

Despite having a large pre-defined non-inferiority margin, our study did not confirm our prespecified hypothesis that DOAC intake at the time of ICH causes a risk of haematoma expansion that is non-inferior compared to no anticoagulation. Instead, 39.9% of patients in the DOAC group compared to 29.7% of non-anticoagulated ICH patients showed HE, albeit with overlapping confidence intervals. The additional multivariate analysis was underpowered but also showed a trend towards an increased risk of haematoma expansion associated with DOAC.

New or increased ventricular extension of the bleeding was observed in 20% of DOAC- compared to 10% in VKA or 14% in non-OAC patients. Although not statistically significant, this difference may be caused by the fact that DOAC affects intrinsic haemostatic mechanisms counteracting secondary haematoma growth [21]. In a healthy brain, dabigatran does not cross the blood–brain barrier, and factor Xa inhibitors are efficiently cleared from the brain by p-glycoprotein efflux pumps [22]. It is speculative as to whether DOAC accumulate with delay after crossing the injured blood–brain barrier.

We did not find a higher proportion of patients with increased haematoma expansion among those taking VKA at the time of ICH compared to non-anticoagulated patients although the majority of VKA patients were effectively anticoagulated based on INR values. This finding contradicts most [23–25], but not all, previous studies [26]. A possible explanation is that 89% of our VKA-ICH patients received PCC for acute reversal. In the INCH trial, rapid reversal of the anticoagulatory effect of VKA with PCC prevented haematoma growth compared to slower-acting fresh frozen plasma [27]. Although an effect of reversal on clinical outcome after VKA-ICH has never been firmly established, current American and European guidelines recommend treatment with PCC plus vitamin K [10, 11].

PCC is also used for haemostatic treatment in DOAC-ICH although evidence for its efficacy is limited [10, 11]. Almost 60% of DOAC-ICH patients available for haematoma expansion analysis in our study received PCC. Consistent with previous studies, our exploratory analysis did not suggest that PCC administration lowers the risk of HE [3, 28]. When the specific DOAC reversal agents idarucizumab and andexanet alfa were evaluated in single-arm clinical trials [29, 30], both agents induced rapid reversal of the thrombin inhibitor dabigatran and factor Xa inhibitors, respectively. The recently published ANNEXA-I trial compared andexanet alfa versus standard treatment showed that andexanet alfa was 13% more haemostatically efficacious than standard treatment – 89% of which received PCC [12]. Only 10.4% of DOAC-ICH patients in the present study received a specific reversal agent, precluding any firm conclusions from the numerically lower rate of haematoma expansion observed in patients treated with andexanet alfa or idarucizumab compared to no reversal or administration of PCC.

Our study has several strengths: It was prospective and concurrently recruited patients with DOACs, VKA and no anticoagulation at the time of ICH following a pre-specified algorithm. Only ICH patients with AF were enrolled, improving the homogeneity among groups. Moreover, RASUNOA-prime applied central neuroimaging analysis following a pre-specified protocol and independent analysis by blinded reviewers. Our study also has limitations: Group enrolment was unevenly distributed, showing a predominance of patients who developed ICH while on DOAC resulting from the less frequent prescription of VKA and the less frequent occurrence of ICH in non-anticoagulated patients with AF, respectively. These imbalances may have also been caused by differences in management among centres with a higher awareness for including ICH patients with DOAC. Nearly 50% of patients without follow-up imaging received early palliative care or had already deceased, precluding them from radiologically haematoma expansion analyses and we cannot rule out that the frequency of haematoma expansion in patients without differs from those with follow-up imaging. Furthermore, the unbalanced size of the groups and the limited availability of follow-up imaging led to a reduction in the power of the primary analysis. With the observed sample size, even if the assumptions of the event rates were correct, the power to detect non-inferiority would be 41%. Finally, participation required informed consent by the patient or the patient’s legal representative at some centres, which may have caused selection bias.

## Conclusions

In this large prospective study, early haematoma volume and neurological deficit were similar in patients with acute ICH with prior DOAC-treatment compared to no oral anticoagulation. The risk of haematoma expansion in DOAC-ICH was not statistically significantly different compared to patients without anticoagulation.

## Supplementary Information

Additional file1 (PDF 413 KB)

## Data Availability

Anonymized data are available upon reasonable request from the corresponding author.

## List of Abbreviations

CT: Computer tomography
DOAC: Direct oral anticoagulant
HE: Haematoma expansion
ICH: Intracerebral haemorrhage
MRI: Magnetic resoncance imaging
PCC: Prothrombin complex concentrate
VKA: Vitamin K antagonist
