# Supplementary material for: Association of oral anticoagulants with risk of brain haemorrhage expansion compared to no-anticoagulation
Source: Neurol Res Pract. 2025 Feb 12;7(1):12. doi: 10.1186/s42466-024-00358-9 (PMC11921975; doi:10.1186/s42466-024-00358-9)
Supplement: Supplementary file 1 — Additional file1 (PDF 413 KB) [file 42466_2024_358_MOESM1_ESM.pdf]

SUPPLEMENTAL MATERIAL

ONLINE SUPPLEMENTAL

Tables

Table S1 Further characteristics of the baseline cohort by anticoagulation scheme

Table S2 Comparison of patients with and without radiological follow-up within 72 h

Table S3 Further characteristics of ‘haematoma expansion analysis cohort’

Table S4 Factors associated with haematoma expansion (Model 1 and model 2 with adjusting variables)

Table S5 Clinical and demographical characteristics of DOAC-ICH patients with radiological follow-up (N = 356) by mode of anticoagulation reversal

Table S6 Anticoagulation reversal in the VKA-ICH subgroup with radiological follow-up

Table S7 Summary of past studies on intracerebral haemorrhage
Part A: No oral anticoagulation
Part B: OAC only
Part C: Mixed (OAC and non-OAC)

List

RASUNOA prime Collaborators

Table S1. *Further characteristics of the baseline cohort by anticoagulation scheme*

	DOAC (N=557)		VKA (N=251)		Non-OAC (N=123)		p-Values	
**Variable**	**N**	**Value**	**N**	**Value**	**N**	**Value**	**DOAC vs. non-OAC**	**VKA vs. non-OAC**
Type of DOAC, n (%)								
Apixaban	577	228 (39.5)	..	..	..	..	..	..
Dabigatran	577	35 (6.1)	..	..	..	..	..	..
Edoxaban	577	62 (10.7)	..	..	..	..	..	..
Rivaroxaban	577	252 (43.7)	..	..	..	..	..	..
Comorbidities, n (%)								
Arterial hypertension	574	538 (93.7)	251	234 (93.2)	121	110 (90.9)	0.262	0.427
Diabetes mellitus	571	142 (24.9)	250	59 (23.6)	121	32 (26.4)	0.716	0.550
Hyperlipidaemia	555	186 (33.5)	233	71 (30.5)	112	30 (26.8)	0.165	0.481
Ischemic heart disease/myocardial infarction	575	177 (30.8)	250	67 (26.8)	121	26 (9.6)	0.041	0.268
Peripheral artery disease	575	31 (5.4)	249	11 (4.4)	120	6 (5.0)	0.8621	0.8026
Previous stroke/TIA	575	148 (25.7)	250	56 (22.4)	121	21 (17.4)	0.051	0.261
Previous ICH	564	20 (3.5)	245	5 (2.0)	114	12 (10.5)	0.001	<0.001
Malignancy	554	70 (12.6)	240	36 (15)	117	23 (19.7)	0.046	0.266
Renal failure	575	48 (8.3)	249	29 (11.6)	121	11 (9.1)	0.790	0.458
Liver insufficiency	575	7 (1.2)	249	6 (2.4)	121	4 (3.3)	0.094	0.618
Smoking	547	39 (7.1)	233	14 (6)	118	10 (8.5)	0.612	0.387
Alcohol abuse	572	24 (4.2)	250	9 (3.6)	122	10 (8.2)	0.063	0.059
Pulmonary embolism	551	19 (3.4)	240	13 (5.4)	118	5 (4.2)	0.676	0.631
Referral, n (%)	577	129 (22.4)	251	63 (25.1)	123	33 (26.8)	0.286	0.719
Length-of-stay, days, median (IQR)	576	9 (4–15)	251	9 (4–15)	123	10 (6–18)	0.035	0.091
Discharge destination, n (%)	401		176		95		0.116	0.003
Home	401	62 (15.5)	176	26 (14.8)	95	12 (12.6)		
Internal hospital transfer	401	8 (2.0)	176	1 (0.6)	95	5 (5.3)		
Secondary hospital	401	56 (14.0)	176	24 (13.6)	95	19 (20.0)		
Rehab	401	241 (60.1)	176	118 (67.0)	95	48 (50.5)		
Nursing facility	401	34 (8.5)	176	7 (4.0)	95	11 (11.6)		

Table S2. *Comparison of patients with and without radiological follow-up within 72 h*

Variable	No FU image		With FU image		p-Value
	N	Value	N	Value	
N (%)	366	366 (100)	585	585 (100)	
Age in years, mean (SD)	366	80.1 (8.14)	585	78.4 (7.95)	0.001
Female sex, n (%)	366	171 (46.7)	585	258 (44.1)	0.430
CHA_2DS_2-Vasc, Median (IQR)	364	4 (3–6)	580	4 (3–5)	0.437
HAS-BLED, Median (IQR)	362	2 (2–3)	577	2 (2–3)	0.027
Coagulation scheme					0.947
DOAC	366	221 (60.4)	585	356 (60.9)	
VKA	366	96 (26.2)	585	155 (26.5)	
Non-OAC	366	49 (13.4)	585	74 (12.6)	
NIHSS at admission, median (IQR)	342	15 (4–25)	563	9 (4–16)	<0.0001
Modified Rankin scale score, median (IQR)					
Pre-stroke	295	1 (0–3)	532	1 (0–2)	0.057
At admission	360	5 (3–5)	572	4 (3–5)	0.003
Discharge	355	5 (3–6)	571	4 (3–5)	<0.0001
Early palliative care, n/N (%)	366	179 (48.9)	585	121 (20.7)	<0.0001
Length-of-stay, days, median (IQR)	365	5 (1–12)	585	11 (7–17)	<0.0001
Death during acute stay	366	171 (46.7)	585	105 (17.9)	<0.0001
Reversal treatment, n (%)					<0.0001
None	317	173 (54.6)	511	124 (24.3)	
PCC	317	131 (41.3)	511	350 (68.5)	
specific*	317	13 (4.1)	511	37 (7.2)	
Baseline haematoma volume, mL, median (IQR)	355	21 (5–79)	568	9 (3–25)	<0.0001

CHA_2DS_2VASc = Cardiac Failure or Dysfunction, Hypertension, Age ≥ 75 years (Doubled), Diabetes, Stroke (Doubled)–Vascular Disease, Age 65–74 Years, and Sex Category (Female); DOAC = direct oral anticoagulant; HAS-BLED = Hypertension, Abnormal Renal/Liver Function, Stroke, Bleeding History or Predisposition, Labile INR [international normalized ratio], Elderly, Drugs/Alcohol Concomitantly; NIHSS = National Institutes of Health Stroke Scale; Non-OAC = no oral anticoagulation; PCC = prothrombincomplex concentrate; VKA = vitamin K antagonists

* Dabigatran: idarucizumab, Factor Xa-inhibitors: andexanet alfa

Table S3. *Further characteristics of 'haematoma expansion analysis cohort'*

	DOAC (N=356)		VKA (N=155)		Non-OAC (N=74)		p-Values	
**Variable**	**N**	**Value**	**N**	**Value**	**N**	**Value**	**DOAC vs. non-OAC**	**VKA vs. non-OAC**
Type of DOAC, n (%)								
Apixaban	356	146 (41)	..	..	..	..	..	..
Dabigatran	356	20 (5.6)	..	..	..	..	..	..
Edoxaban	356	40 (11.2)	..	..	..	..	..	..
Rivaroxaban	356	150 (42.1)	..	..	..	..	..	..
Concomitant antiplatelet therapy, n (%)	351	31 (8.8)	155	11 (7.1)	71	32 (45.1)	<0.0001	<0.0001
Reversal treatment, n (%)								
None	356	107 (30.1)	155	17 (11)	..	..	..	..
PCC	356	212 (59.6)	155	138 (89)	..	..	..	..
Specific[†]	356	37 (10.4)	155	0 (0)	..	..	..	..
Reversal treatment, n (%)								
Systolic blood pressure at admission, mmHg, median (IQR)	338	168 (150–190)	147	166 (148–189)	72	167 (150–200)	0.928	0.357
Systolic blood pressure at 24 hours after admission, mmHg, median (IQR)	306	135 (120–147)	128	135 (120–148)	69	133 (120–148)	0.793	0.908
Comorbidities, n (%)								
Arterial hypertension	355	340 (95.8)	155	147 (94.8)	72	67 (93.1)	0.319	0.591
Diabetes mellitus	355	96 (27)	155	37 (23.9)	72	15 (20.8)	0.273	0.612
Hyperlipidaemia	344	122 (35.5)	144	42 (29.2)	68	20 (29.4)	0.337	0.971
Ischemic heart disease/myocardial infarction	356	109 (30.6)	155	43 (27.7)	72	15 (20.8)	0.095	0.267
Peripheral artery disease	356	15 (4.2)	154	4 (2.6)	72	4 (5.6)	0.614	0.262
Previous stroke/TIA	356	89 (25.0)	155	37 (23.9)	72	12 (16.7)	0.129	0.22
Previous ICH	356	10 (2.8)	151	3 (1.9)	68	9 (13.3)	<0.001	<0.001
Malignancy	345	42 (12.2)	150	18 (12)	70	10 (14.3)	0.627	0.636
Renal failure	356	24 (6.7)	154	12 (7.8)	72	6 (8.3)	0.630	0.889
Liver insufficiency	356	5 (1.4)	154	3 (1.9)	72	3 (4.2)	0.115	0.334
Smoking	339	28 (8.3)	144	10 (6.9)	70	6 (8.6)	0.931	0.671
Alcohol abuse	354	18 (5.1)	154	5 (3.2)	73	5 (6.8)	0.543	0.217
Pulmonary embolism	341	11 (3.2)	149	9 (6)	71	2 (2.8)	0.858	0.305

Length-of-stay, days, median (IQR)	356	11 (7–17)	155	11 (6–17)	74	13 (8–18)	0.213	0.175
Discharge destination, n (%)	293						0.435	0.010
Home	293	41 (14.0)	122	15 (12.3)	63	7 (11.1)		
Internal hospital transfer	293	6 (2.1)	122	0 (0)	63	3 (4.8)		
Secondary hospital	293	43 (14.7)	122	17 (13.9)	63	13 (20.6)		
Rehab	293	181 (61.8)	122	87 (71.3)	63	34 (54.0)		
Nursing facility	293	22 (7.5)	122	3 (2.5)	63	6 (9.5)		
Onset to first brain imaging‡, hours, median (IQR)	338	2 (1–5)	146	2 (1–5)	67	2 (1–6)	0.280	0.773
Admission to follow-up imaging showing maximum haematoma expansion, hours, median (IQR)	142	18 (8–31)	47	18 (10–28)	22	24 (18–48)	0.039	0.069

CHA_2DS_2VASc = Cardiac Failure or Dysfunction, Hypertension, Age ≥ 75 years (Doubled), Diabetes, Stroke (Doubled)–Vascular Disease, Age 65–74 Years, and Sex Category (Female); DOAC = direct oral anticoagulant; HAS-BLED = Hypertension, Abnormal Renal/Liver Function, Stroke, Bleeding History or Predisposition, Labile INR [international normalized ratio], Elderly, Drugs/Alcohol Concomitantly; NIHSS = National Institutes of Health Stroke Scale; No-OAC = no oral anticoagulation; PCC = prothrombincomplex concentrate; VKA = vitamin K antagonists

† Dabigatran: idarucizumab, Factor Xa-inhibitors: andexanet alfa
‡ Onset: symptom onset, in case of unknown onset, last-seen-well

Table S4. *Factors associated with haematoma expansion (Model 1 and model 2 with adjusting variables)*

**Model 1**					
	**N**	**Univariable OR (95%-CI)**	**p-value**	**Multivariable (N = 412) OR (95%-CI)**	**p-value**
Age in categories, years	585		0.981		0.976
18–64		1		1	
65–74		1.02 (0.45–2.30)		0.86 (0.29–2.51)	
75–84		1.10 (0.51–2.37)		0.88 (0.31–2.44)	
≥ 85		1.11 (0.50–2.49)		0.96 (0.33–2.79)	
Male vs. female	585	1.06 (0.76-1.50)	0.722	0.98 (0.93-1.02)	0.613
Onset to first brain imaging, hours	543	0.93 (0.90–0.97)	<0.001	0.95 (0.91–0.99)	0.020
Baseline haematoma volume, mL (log)	568	1.00 (0.89–1.12)	0.973	1.26 (1.04–1.51)	0.017
mGraeb admission	585	0.98 (0.94-1.01)	0.126	0.98 (0.93-1.02)	0.280
Initial systolic blood pressure >160 mm Hg	557	0.83 (0.59–1.17)	0.290	0.77 (0.51–1.17)	0.225
Concomitant antiplatelet therapy	577	0.90 (0.54–1.51)	0.688	1.01 (0.51–2.06)	0.989
ICH location	503		0.679		0.115
Deep		1		1	
Lobar		1.21 (0.79–1.85)		1.89 (1.04–3.46)	
Deep + lobar		1.11 (0.70–1.78)		1.25 (0.72–2.15)	
mRS pre- stroke	585		0.230		0.548
0–3		1		1	
4–5		0.69 (0.45–1.08)		0.77 (0.45–1.33)	
not documented		1.09 (0.61–1.96)		1.18 (0.54–2.56)	
Glucose at admission, mg/dL (log)	548	0.62 (0.34–1.13)	0.119	0.43 (0.20–0.92)	0.029
**Model 2: Effects of DOAC and VKA in two separated models, adjusted for logit of model 1**					
		**Adjusted OR (95%-CI)**	**p-value**		
DOAC vs. no-OAC		1.58 (0.81–3.08)	0.180		
VKA vs. no-OAC		1.02 (0.48–2.21)	0.952		

Table S5. *Clinical and demographical characteristics of DOAC-ICH patients with radiological follow-up (N = 356) by mode of anticoagulation reversal*

	No Antagonization		PCC		Specific Antagonization†		
	**N**	**Value**	**N**	**Value**	**N**	**Value**	**p-Value**
N		107 (30.1%)		212 (59.6%)		37 (10.4%)	..
Age categories, years	107		212		37		0.567
18–64		7 (6.5)		10 (4.7)		0 (0.0)	
65–74		28 (26.2)		54 (25.5)		8 (21.6)	
75–84		51 (47.7)		102 (48.1)		17 (46.0)	
≥ 85		21 (19.6)		46 (21.7)		12 (32.4)	
Female sex, n (%)	107	46 (43.0)	212	103 (48.6%)	37	19 (51.4)	0.554
Palliative care during acute stay, n (%)	107	16 (15.0)	212	48 (22.6%)	37	11 (29.7)	0.111
NIHSS at admission, median (IQR)	105	6 (2–12)	201	10 (5–17)	37	7 (4–13)	0.127
mRS pre-stroke	107		212		37		0.206
0–3		82 (76.6)		151 (71.2)		24 (64.9)	
4–5		19 (17.8)		47 (22.2)		7 (18.9)	
Not documented		6 (5.5)		14 (6.6)		6 (16.2)	
mRS admission > 2	104	80 (76.9)	205	185 (90.2)	37	31 (83.8)	0.007
mRS at discharge, median (IQR)	102	3 (3–5)	206	5 (3–5)	37	4 (3–5)	<0.001
Death during acute stay, n (%)	107	13 (12.2)	212	40 (18.9)	37	9 (24.3)	0.165
HAS-BLED >2	107	36 (33.6)	210	58 (27.6)	37	11 (29.7)	0.540
CHA_2DS_2-VASc-Score	107	5 (3–6)	212	4 (3–5)	37	5 (3–5)	0.404
SBP at admission, mmHg, mean (SD)	101	166 (31)	203	170 (34)	34	167 (28)	0.413
Symptom onset to imaging in hours, median (IQR)	92	2 (1–6)	200	2 (1–4)	35	2 (1–5)	0.467
Haematoma volume, median (IQR)	107	6.1 (2.1–23.5)	212	9.7 (4.5–27.5)	37	11.3 (2.6–18.0)	0.082
ICH location, n (%)	91		184		31		0.080
Deep		42 (46.2)		56 (30.4)		9 (29.0)	
Lobar		26 (28.6)		80 (43.5)		13 (41.9)	
Deep and lobar		23 (25.3)		48 (26.1)		9 (29.0)	
Substantial haematoma expansion, n (%)	107	41 (38.3)	212	89 (42.0)	37	12 (32.4)	0.508

CHA_2DS_2VASc = Cardiac Failure or Dysfunction, Hypertension, Age ≥ 75 years (Doubled), Diabetes, Stroke (Doubled)–Vascular Disease, Age 65–74 Years, and Sex Category (Female); DOAC = direct oral anticoagulant; HAS-BLED = Hypertension, Abnormal Renal/Liver Function, Stroke, Bleeding History or Predisposition, Labile INR [international normalized ratio], Elderly, Drugs/Alcohol Concomitantly; mRS = modified Rankin scale score; NIHSS = National Institutes of Health Stroke Scale; SBP = Systolic blood pressure.

† Dabigatran: idarucizumab, Factor Xa-inhibitors: andexanet alfa

Table S6. *Anticoagulation reversal in the VKA-ICH subgroup with radiological follow-up*

	No Antagonization		PCC		
	**N**	**Values**	**N**	**Values**	**p-Value**
N (%)		17 (11.0)		137 (89.0)	
Age categories, years	17		138		0.092
18–64		3 (17.6)		6 (4.4)	
65–74		1 (5.9)		25 (18.1)	
75–84		8 (47.1)		76 (55.1)	
≥ 85		5 (29.4)		31 (22.5)	
Sex (female), n (%)	17	10 (58.8)	138	48 (34.8)	0.053
NIHSS at admission, median (IQR)	15	6 (1–26)	132	9 (4–18)	0.261
mRS pre-stroke	17		138		0.983
0–3		12 (70.6)		100 (72.5)	
4–5		3 (17.7)		22 (15.9)	
not documented		2 (11.8)		16 (11.6)	
mRS admission >2	17	11 (64.7)	136	119 (87.5)	0.013
Haematoma volume, mL median (IQR)	17	7.5 (4.2–50.0)	137	9.7 (4.6–22.5)	0.879
Substantial haematoma expansion, n (%)	17	4 (23.5)	138	43 (31.2)	0.518
mRS at discharge, Median (IQR)	17	3 (2-6)	138	3 (3-5)	0.100
mRS >2		9 (52.9)		116 (84.7)	0.002
Death during acute stay	17	5 (29.4)	138	27 (19.6)	0.344
INR at admission	16		133		0.127
INR < 2		7 (43.8)		28 (21.1)	
INR 2–3		6 (37.5)		66 (48.2)	
INR ≥ 3		3 (18.8)		39 (29.3)	

mRS = modified Rankin scale score; NIHSS = National Institutes of Health Stroke Scale; PCC = prothrombincomplex concentrate; VKA = vitamin K antagonists

Table S7 Summary of past studies on intracerebral haemorrhage

Part A: No oral anticoagulation
Part B: OAC only
Part C: Mixed (OAC and non-OAC)

PART A	NO ORAL ANTICOAGULATION (Non-OAC)			
Reference	Kazui et al.[1]	Brott et al.[2]	Davis et al.[3]	Mayer et al.[4]
Year	1996	1997	2006	2008
Study Type	Retrospective analysis	Prospective observational	Pooled meta-analysis	RCT
N	204	103	218	268 (placebo-arm)
Age, mean, years	64	63	66	65
Women, %	37.7	36.0	41.7	37.0
Oral Anticoagulation, No. (%)	0 (0)	0 (0)	0 (0)	0 (0)
Reversal therapy	-	-		
Haematoma volume (mL)			(placebo-arms)	(placebo-arm)
Median (IQR)	NR	NR	NR	NR
Mean (SD)	20.1 (18.0)	26 (29)	25.3 (NR)	22 (24)
Haematoma Expansion				
Predefined time frame	0–120 h	0–20h	24 h	21–48 h
Definition of significant expansion	> 12.5 ml or > 40%	≥ 33%	> 33%	-
Proportion of patients with significant expansion	19.6%	38.0%	31.6%	NR[e]

PART B	ORAL ANTICOAGULATION ONLY (Studies before 2018)						
**Reference**	Kuramatsu et al.[5]	Purrucker et al.[6]	Steiner et al.[7]	Connolly et al.[8]	Wilson et al.[9]	Tsivgoulis et al.[10]	Melmed et al.[11]
**Year**	2015	2016	2016	2016	2017	2017	2017
**Study Type**	Retrospective cohort study (VKA)	Prospective observational (DOAC)	RCT<br><br>(VKA)	Prospective observational (Factor-Xa antagonists incl. enoxaparin)	Pooled analysis	Multicenter cross-sectional + systematic review	Retrospective single-center
**N**	853	61	50	14 (ICH only)	500	161	27
**Age, mean, years**	74	76	76	NR	Median: 80	76	VKA 77<br>DOAC 79
**Women, %**	37.9	41	38	NR	45 (DOAC), 51 (VKA)	42	56
**Oral Anticoagulation, Type, No. (%)**	VKA 853 (100)	DOAC 61 (100)	VKA 50 (100)	NR	DOAC 97 (19), VKA 403 (81)	DOAC 47 (29)<br>VKA 114 (71)	DOAC 9<br>VKA 18
**Reversal therapy**	VK+/- PCC+-FFP	PCC (35 (57%))	PCC vs. FFP	Andexanet alfa (100%)	PCC (VKA 286/365 (78%); DOAC 35/85 (41%))	PCC (VKA 82 (72%), DOAC 16 (34%))<br><br>FFP (VKA 31 (27%); DOAC 3 (6%)))<br>Idarucizumab (DOAC 1 (2%))	PCC (VKA 4 (22%); DOAC 4 (44%))<br><br>FFP (VKA 6 (33)<br>Factor IX (VKA 10 (56%), DOAC 2 (22%))

**Haematoma volume (mL)**							
**Median (IQR)**	19.3 (6.9 – 52.8)	10.8 (4.0 – 30.0)	FFP: 13.2 (0.2 – 43.9)<br><br>PCC: 13.0 (0.6 – 78.1)	NR	VKA 10.6 (4.0-27.9)<br>DOAC 14.4 (3.6-38.4);	VKA 24.3 (11 – 58.8)<br>DOAC 12.8 (4–40)	NR
**Mean (SD)**	NR	23.7 (31.3)	NR	NR (8/14 ≤ 10 ml; 6/14 11 to 60 mL)	NR	NR	(not specified, likely mean reported):<br>VKA 24 (SD nr.)<br>DOAC 28 (SD nr.)
**Haematoma Expansion**							
**Predefined time frame analysed**	NR	3–72 h	3 h, 24 h, 72 h	1 h, 12 h	< 72 h	24 h	24 h
**Definition of significant expansion**	≥ 33%	≥ 6 ml or ≥ 33%	≥ 33% or death[f]	“effective hemostasis: ≤ 20%; good hemostasis: > 20% ≤ 35%”	≥ 6 ml or ≥ 33%	> 12.5 ml or > 33%	≥ 12 mL or ≥ 33%
**Proportion of patients with significant expansion**	36%	38%	24 h:<br><br>FFP: 60%<br><br>PCC: 30%	NR<br><br>(intracranial hemorrhage, effective/good hemostasis: 80% (56–94%)	VKA 34%<br>DOAC 40%,	VKA 37.1%<br>DOAC 23.1%	VKA 50%<br>DOAC 44%

PART B	ORAL ANTICOAGULATION ONLY (Studies 2018 – 2024)						
**Reference**	Gerner et al.[12]	Lioutas et al.[13]	Demchuk et al.[14]	Rowe et al.[15]	Polymeris et al.[16]	Ip et al.[17]	Connolly et al.[18]
**Year**	2018	2018	2021	2023	2023	2024	2024
**Study Type**	Retrospective, multicenter	Prospective, multicenter	RCT (Subanalysis / ANNEXA-4)	Retrospective, single-center	RCT (TICH-DOAC)	Retrospective, multicenter	RCT (ANNEXA-I)
**N**	190 (146 HE analysis)	196 (pmRS < 2 only)	99 (Efficacy analysis, spontaneous ICH)	40	63	232 (after PSM: 182)	452 (efficacy analysis)
**Age, mean, years**	77 (with HE), 78 (without)	VKA 72<br>DOAC 75	78	75	Median 82	77	79
**Women, %**	36.7 (with HE), 52.6% without)	VKA 98 (27)<br>DOAC 28 (45)	47 (48%)	16 (40)	25 (40%)	44%	Andexanet: 42%<br>Usual care: 50%
**Oral Anticoagulation, Type, No. (%)**	DOAC 190 (100)	VKA 134 (68)<br>DOAC 62 (32)	DOAC 99 (100) (FXa-Inh. only)	DOAC 40 (100) (FXa-Inh. only)	DOAC 63 (100%) (FXa-Inh. only)	DOAC 232 (100%)	DOAC 452 (100) (FXa-Inh. only)
**Reversal therapy**	PCC 108/146* (74%)<br><br>(*according to table 1, according to figure 1: 103)	PCC (VKA 63%; DOAC 44%); FFP (VLA 20%, DOAC 6%); Idarucziumab (DOAC 2%), Andexanet alfa (DOAC 2%)	Andexanet alfa (100%)	PCC (40 (100%))	PCC 41 (65%)<br>TXA 32 (51%)<br>[TXA+PCC 22/32]	PCC (85/182 (47%))	Anexanet alfa 224 (50)

**Haematoma volume (mL)**							
**Median (IQR)**	13.3 (64.–30.2) with HE; 9.3 (3.4–28.9) without HE	VKA 19.5 (6.6 – 52.0)<br>DOAC 13.8 (2.5 – 37.6)	9.4 (3.3 – 20.8)	NR	11.5 (4.8–27.4)	PCC: 17.6 (4.2 – 52.2)<br>No-PCC: 24.5 (2.6 – 61.1)	Andexanet: 10.5 (4.1–24.9)<br>Usual care: 9 (3.1–22.8)
**Mean (SD)**	NR	NR	NR	10.3 ± 15 mL	NR	NR	NR
**Haematoma Expansion**							
**Predefined time frame analysed**	Not defined	24 h	12 h	> 6 and < 40 h	24 h	3 to 72 h	12 h
**Definition of significant expansion**	> 33%	> 12.5 ml or > 33%	> 35%	> 6 ml or > 33%	≥ 6 ml or ≥ 33%	> 6 ml or > 33%	> 35%
**Proportion of patients with significant expansion**	33.6%	VKA 37.4%<br>DOAC 17%	21/98 (21.4%)	15.7%	41/63 (41%)	FU-imaging available in 96 pts<br><br>PCC: 13 (27%)<br>No-PCC: 13 (27%)	Andexanet:<br><br>50/215 (23.2%)<br>Usual care: 75/212 (35.4%)

PART C	MIXED - ORAL ANTICOAGULATION and No ORAL ANTICOAGULATION (OAC + Non-OAC)							
**Reference**	Flibotte et al.[19]	Flaherty et al.[20]	Cucchiara et al.[21]	Huhtakangas et al.[22]	Horstmann et al.[23]	Sembill et al.[24]	Siepen et al.[25]	**This study**
**Year**	2004	2008	2008	2011	2013	2020	2024	2024
**Study Type**	Prospective cohort study	Retrospective cohort study	RCT substudy	Retrospective observational	Prospective observational	Pooled analysis of mainly retrospective data; non-OAC from single registry	Pooled analysis of national stroke registries	Prospective multicenter
**N**	183 (70 HE analysis)	258	303 (285 HE analysis)	982	206 (152 HE analysis)	1954 (supratenorial ICH)	11349	951
**Age, mean, years**	76	69	NR (OAC: 75)	69	74[b]	68–78* (* range of medians)	74	79
**Women, %**	NR	54.3	33.9	46.2	47.6	40.6	47.6	45.1
**Oral Anticoagulation, Type, No. (%)**	VKA 42 (23.0)	VKA 51 (19.8)	VKA 21 (6.9)	VKA 182 (18.5)	VKA 51 (24.8)	VKA 1186 (60.7) DOAC 107 (5.5) Non-OAC 661 (33.8)	VKA 1491 (13.1) DOAC 1205 (10.6) Non-OAC 8653 (76.2)	VKA 251 (26.3) DOAC 557 (58.6) Non-OAC 123 (12.9)
**Reversal therapy**	VK + FFP	-	not specified	partly: VK+PCC	VK+PCC or FFP	VKA: PCC 94%; FFP 6.1% DOAC: PCC 74.8%	NR	VKA: 193 (76.9%) DOAC: Specific: 50 (8.7%); PCC 288 (49.9%)

**Haematoma volume (mL)**								
**Median (IQR)**	NR	NR	Non-OAC: 14.4 (7.9 – 30.9)<br><br>OAC: 30.6 (7.4 – 70.1)	NR	Non-OAC 14.3 (4.9 – 35.7)<br><br>OAC: 20.0 (8.3 – 48.8)	VKA: 9.7–20.9 DOAC: 9.2.–22.2 Non-OAC: 8.8–17.9 (Range of medians)	NR	VKA: 11 (5–43) DOAC: 13 (4–38) Non-OAC: 10 (3–30)
**Mean (SD)**	NR	[d]	NR	Non-OAC: 29.6 (37.0)<br><br>OAC: 47.8 (58.0)	Non-OAC: 26.4 (31.7)<br><br>OAC: 31.5 (30.2)	NR	NR	VKA: 31.8 (42.7) DOAC: 28.4 (37.2) Non-OAC: 26.7 (37.5)
**Haematoma Expansion**								
**Predefined time frame analysed**	0–7 d	-	0–72 h	NR	24–48 h	Not defined	n.a.	≤ 72 h
**Definition of significant expansion**	≥ 33%	NR	≥ 33%	NR	≥ 6 ml or ≥ 33%	≥ 6 ml or ≥ 33%		≥ 6.5 ml or ≥ 33% or new ventricular extension/mGraeb+≥ 2 Pt.
**Proportion of patients with significant expansion**	Non-OAC 23%<br><br>OAC: 54%	NR	Non-OAC: 26%<br><br>OAC: 56%	NR	non-OAC: 11.7%<br><br>OAC: 12.5%	VKA: 36.4% DOAC: 30.8% Non-OAC: 11.3%		VKA: 30.3% DOAC: 39.9% Non-OAC: 29.7%

Supplementary References

1. Kazui S, Naritomi H, Yamamoto H, et al. Enlargement of spontaneous intracerebral hemorrhage. Incidence and time course. *Stroke* 1996;27(10):1783-7. doi: 10.1161/01.str.27.10.1783.
2. Brott T, Broderick J, Kothari R, et al. Early hemorrhage growth in patients with intracerebral hemorrhage. *Stroke* 1997;28(1):1-5. doi: 10.1161/01.str.28.1.1.
3. Davis SM, Broderick J, Hennerici M, et al. Hematoma growth is a determinant of mortality and poor outcome after intracerebral hemorrhage. *Neurology* 2006;66(8):1175-81. doi: 10.1212/01.wnl.0000208408.98482.99.
4. Mayer SA, Brun NC, Begtrup K, et al. Efficacy and safety of recombinant activated factor VII for acute intracerebral hemorrhage. *N Engl J Med* 2008;358(20):2127-37. doi: 10.1056/NEJMoa0707534.
5. Kuramatsu JB, Gerner ST, Schellinger PD, et al. Anticoagulant reversal, blood pressure levels, and anticoagulant resumption in patients with anticoagulation-related intracerebral hemorrhage. *JAMA* 2015;313(8):824-36. doi: 10.1001/jama.2015.0846.
6. Purrucker JC, Haas K, Rizos T, et al. Early Clinical and Radiological Course, Management, and Outcome of Intracerebral Hemorrhage Related to New Oral Anticoagulants. *JAMA Neurol* 2016;73(2):169-77. doi: 10.1001/jamaneurol.2015.3682.
7. Steiner T, Poli S, Griebe M, et al. Fresh frozen plasma versus prothrombin complex concentrate in patients with intracranial haemorrhage related to vitamin K antagonists (INCH): a randomised trial. *Lancet Neurol* 2016;15(6):566-73. doi: 10.1016/S1474-4422(16)00110-1.
8. Connolly SJ, Milling TJ, Jr., Eikelboom JW, et al. Andexanet Alfa for Acute Major Bleeding Associated with Factor Xa Inhibitors. *N Engl J Med* 2016;375(12):1131-41. doi: 10.1056/NEJMoa1607887.
9. Wilson D, Seiffge DJ, Traenka C, et al. Outcome of intracerebral hemorrhage associated with different oral anticoagulants. *Neurology* 2017;88(18):1693-700. doi: 10.1212/WNL.0000000000003886.
10. Tsivgoulis G, Wilson D, Katsanos AH, et al. Neuroimaging and clinical outcomes of oral anticoagulant-associated intracerebral hemorrhage. *Ann Neurol* 2018;84(5):694-704. doi: 10.1002/ana.25342.
11. Melmed KR, Lyden P, Gellada N, et al. Intracerebral Hemorrhagic Expansion Occurs in Patients Using Non-Vitamin K Antagonist Oral Anticoagulants Comparable with Patients Using Warfarin. *J Stroke Cerebrovasc Dis* 2017;26(8):1874-82. doi: 10.1016/j.jstrokecerebrovasdis.2017.04.025.
12. Gerner ST, Kuramatsu JB, Sembill JA, et al. Characteristics in Non-Vitamin K Antagonist Oral Anticoagulant-Related Intracerebral Hemorrhage. *Stroke* 2019;50(6):1392-402. doi: 10.1161/STROKEAHA.118.023492.
13. Lioutas VA, Goyal N, Katsanos AH, et al. Clinical Outcomes and Neuroimaging Profiles in Nondisabled Patients With Anticoagulant-Related Intracerebral Hemorrhage. *Stroke* 2018;49(10):2309-16. doi: 10.1161/STROKEAHA.118.021979.
14. Demchuk AM, Yue P, Zotova E, et al. Hemostatic Efficacy and Anti-FXa (Factor Xa) Reversal With Andexanet Alfa in Intracranial Hemorrhage: ANNEXA-4 Substudy. *Stroke* 2021;52(6):2096-105. doi: 10.1161/STROKEAHA.120.030565.
15. Rowe AS, Hamilton LA, Barber JA, et al. Activated Prothrombin Complex Concentrates for the Treatment of Factor Xa Inhibitor-Associated Spontaneous Intracerebral Hemorrhage. *J Pharm Technol* 2023;39(6):286-90. doi: 10.1177/87551225231204749.
16. Polymeris AA, Karwacki GM, Siepen BM, et al. Tranexamic Acid for Intracerebral Hemorrhage in Patients on Non-Vitamin K Antagonist Oral Anticoagulants (TICH-NOAC): A Multicenter, Randomized, Placebo-Controlled, Phase 2 Trial. *Stroke* 2023;54(9):2223-34. doi: 10.1161/STROKEAHA.123.042866.

17. Ip B, Pan S, Yuan Z, et al. Prothrombin Complex Concentrate vs Conservative Management in ICH Associated With Direct Oral Anticoagulants. *JAMA Netw Open* 2024;7(2):e2354916. doi: 10.1001/jamanetworkopen.2023.54916.
18. Connolly SJ, Sharma M, Cohen AT, et al. Andexanet for Factor Xa Inhibitor-Associated Acute Intracerebral Hemorrhage. *N Engl J Med* 2024;390(19):1745-55. doi: 10.1056/NEJMoa2313040.
19. Flibotte JJ, Hagan N, O'Donnell J, et al. Warfarin, hematoma expansion, and outcome of intracerebral hemorrhage. *Neurology* 2004;63(6):1059-64. doi: 10.1212/01.wnl.0000138428.40673.83.
20. Flaherty ML, Tao H, Haverbusch M, et al. Warfarin use leads to larger intracerebral hematomas. *Neurology* 2008;71(14):1084-9. doi: 10.1212/01.wnl.0000326895.58992.27.
21. Cucchiara B, Messe S, Sansing L, et al. Hematoma growth in oral anticoagulant related intracerebral hemorrhage. *Stroke* 2008;39(11):2993-6. doi: 10.1161/STROKEAHA.108.520668.
22. Huhtakangas J, Tetri S, Juvela S, et al. Effect of increased warfarin use on warfarin-related cerebral hemorrhage: a longitudinal population-based study. *Stroke* 2011;42(9):2431-5. doi: 10.1161/STROKEAHA.111.615260.
23. Horstmann S, Rizos T, Lauseker M, et al. Intracerebral hemorrhage during anticoagulation with vitamin K antagonists: a consecutive observational study. *J Neurol* 2013;260(8):2046-51. doi: 10.1007/s00415-013-6939-6.
24. Sembill JA, Kuramatsu JB, Gerner ST, et al. Hematoma enlargement characteristics in deep versus lobar intracerebral hemorrhage. *Ann Clin Transl Neurol* 2020;7(3):363-74. doi: 10.1002/acn3.51001.
25. Siepen BM, Forfang E, Branca M, et al. Intracerebral haemorrhage in patients taking different types of oral anticoagulants: a pooled individual patient data analysis from two national stroke registries. *Stroke Vasc Neurol* 2024 doi: 10.1136/svn-2023-002813.

COLLABORATORS

Further collaborators for the RASUNOA prime ICH substudy

Affiliation*	Name
Heidelberg University Hospital	Solveig Horstmann<br>Alexandra Krauß<br>Caroline Renninger<br>Peter Ringleb
University Hospital Aachen	Arno Reich
SLK-Klinken Heilbronn	Eve Kohler
University Hospital Frankfurt a. M.	Erendira Boss<br>Jan Hendrik Schaefer
Johannes Wesling Klinikum Minden	Marc Pflug
University Medicine Greifswald	Bettina von Sarnowski
Hannover Medical School	Gerrit Maximilian Große<br>Johanna Ernst<br>Karin Weißenborn<br>Ramona Schupper<br>Hans Worthmann
University Hospital Schleswig-Holstein, Lübeck	Susanne Riebau
Vivantes Klinikum Neukölln Berlin	Olaf Crome<br>Boris Dimitrijeski<br>Jens Offermann
Charité Berlin, Department for Neurology with experimental Neurology and Center for Stroke Research Berlin (CSB)	Ida Rangus
Klinikum Stuttgart	Elisabeth Schmid
University Hospital Tübingen	Khouloud Poli<br>Johannes Tünnerhoff
University Hospital Leipzig	Dominik Michalski<br>Johann O. Pelz
University Hospital Gießen	Martin Juenemann<br>Tobias Braun
Martin-Luther-University of Halle-Wittenberg	Tobias J. Müller<br>Katja Wartenberg
University Hospital Schleswig-Holstein, Kiel	Andreas Binder<br>Johannes Meyne
Klinikum Rechts der Isar, München	Benno Ikenberg<br>Johanna Härtl
Herz-Jesu-Hospital Hiltrup, Münster	Michael Ohms<br>Sebasitan Edelbusch
University Hospital Mannheim	Marc Fatar<br>Angelika Alonso
Klinikum Nürnberg-Süd, Paracelsis Medical Clinic, Nürnberg	Martin Nückel<br>Frank Erbguth
University Hospital Würzburg	Alexandra Grau<br>Udo Selig
University Hospital Münster	Rainer Dziewas<br>Jens Minnerup
University Hospital Dresden	Kristian Barlinn<br>Kathrin Haase

University Hospital Hamburg-Eppendorf, Hamburg	Götz Thomalla<br>Milani Deb-Chatterji
Juliusspital Würzburg	Mathias Mäurer<br>Mathias Pfau
Asklepios Klinik Altona, Hamburg	Peter Michels<br>Zoran Vukovic
University Hospital Mainz	Timo Uphaus<br>Klaus Gröschel<br>Sonja Gröschel<br>Marianne Hahn
Leopoldina-Krankenhaus Schweinfurth	Joannes Mühler<br>Klaus Dötter
Helios Dr. Horst Schmidt Kliniken Wiesbaden	Michaela Wagner-Heck<br>Frank Arne Wollenweber
University Hospital Düsseldorf	Sebastian Jander<br>John-Ih Lee
Evangelisches Krankenhaus Bielefeld	Wolf-Rüdiger Schäbitz<br>Inken Piehl
Krankenhaus der Barmherzigen Brüder Eisenstadt, Austria	Michael Frattner<br>Dimitre Staykov
Klinikum Ludwigshafen	Christian Urbanek<br>Sabine Schröder
Evangelisches Klinikum Herne	Sylke Düllberg-Boden
Nordwest-Krankenhaus Sanderbusch	Pawel Kermer<br>Matthias Kaste
Asklepios Klinik Wandsbek, Hamburg	Lars Marquardt<br>Haiko Kazarians
Universitätsklinikum Essen	Christoph Kleinschnitz<br>Peter Kraft
Martha-Maria Hospital Halle	Frank Hoffmann<br>Andrea Kraft
Asklepios Fachklinik Teupitz	Jürgen Hartmut Faiss
Klinikum Dortmund	Gernot Reimann<br>Michael Schwarz
Frankfurt Hoechst, Frankfurt a. M.	Thorsten Steiner
University Hospital Jena	Albrecht Günther
Hospital Nordwest, Frankfurt a. M.	Uta Meyding Lamadé<br>Matthias W. Lorenz

* Germany, if not otherwise indicated. Principal- and co investigators who enrolled at least 1 patient in the RASUNOA-prime ICH substudy are listed.
